# The treatment of depression — searching for new ideas

**DOI:** 10.3389/fphar.2022.988648

**Published:** 2022-10-07

**Authors:** Katarzyna Stachowicz, Magdalena Sowa-Kućma

**Affiliations:** ^1^ Department of Neurobiology, Maj Institute of Pharmacology, Polish Academy of Sciences, Kraków, Poland; ^2^ Department of Human Physiology, Institute of Medical Sciences, Medical College of Rzeszow University, Rzeszow, Poland; ^3^ Centre for Innovative Research in Medical and Natural Sciences, Medical College of Rzeszow University, Rzeszow, Poland

**Keywords:** depression, antidepressants, new target, glutamate receptors, DSCAM

## Abstract

Depression is a severe mental health problem that affects people regardless of social status or education, is associated with changes in mood and behavior, and can result in a suicide attempt. Therapy of depressive disorders is based mainly on drugs discovered in the 1960s and early 1970s. Selective serotonin reuptake inhibitors (SSRIs) and serotonin-norepinephrine reuptake inhibitors (SNRIs) are frontline pharmacological strategies for the medical treatment of depression. In addition, approved by FDA in 2019, esketamine [as nasal spray; N-methyl-D-aspartate (NMDA) receptors antagonist with additional effects on α-Amino-3-hydroxy-5-methyl-4-isoxazolepropionic acid (AMPA) receptors, hyperpolarization-activated cyclic nucleotide-gated (HCN) channels, L-type voltage-dependent calcium channel (L-VDCC), opioid receptors, and monoaminergic receptors] is an essential compound in suicide and drug-resistant depression. However, the treatment of depression is burdened with severe side effects, and in many cases, it is ineffective. An equally important issue is the choice of antidepressant therapy in people with comorbid somatic diseases, for example, due to possible interactions with the patient's other drugs. Therefore, there is a great need for new antidepressants with different mechanisms of action and the need to refine the search for new substances. The purpose of this review was to discuss new research directions and new trends that dominate laboratories worldwide. We have reviewed the literature to present new points on the pharmacological target of substances with antidepressant activity. In addition, we propose a new perspective on depressive therapies.

## Background to depression

### The disease of emotions and motivation

**FIGURE 1:**
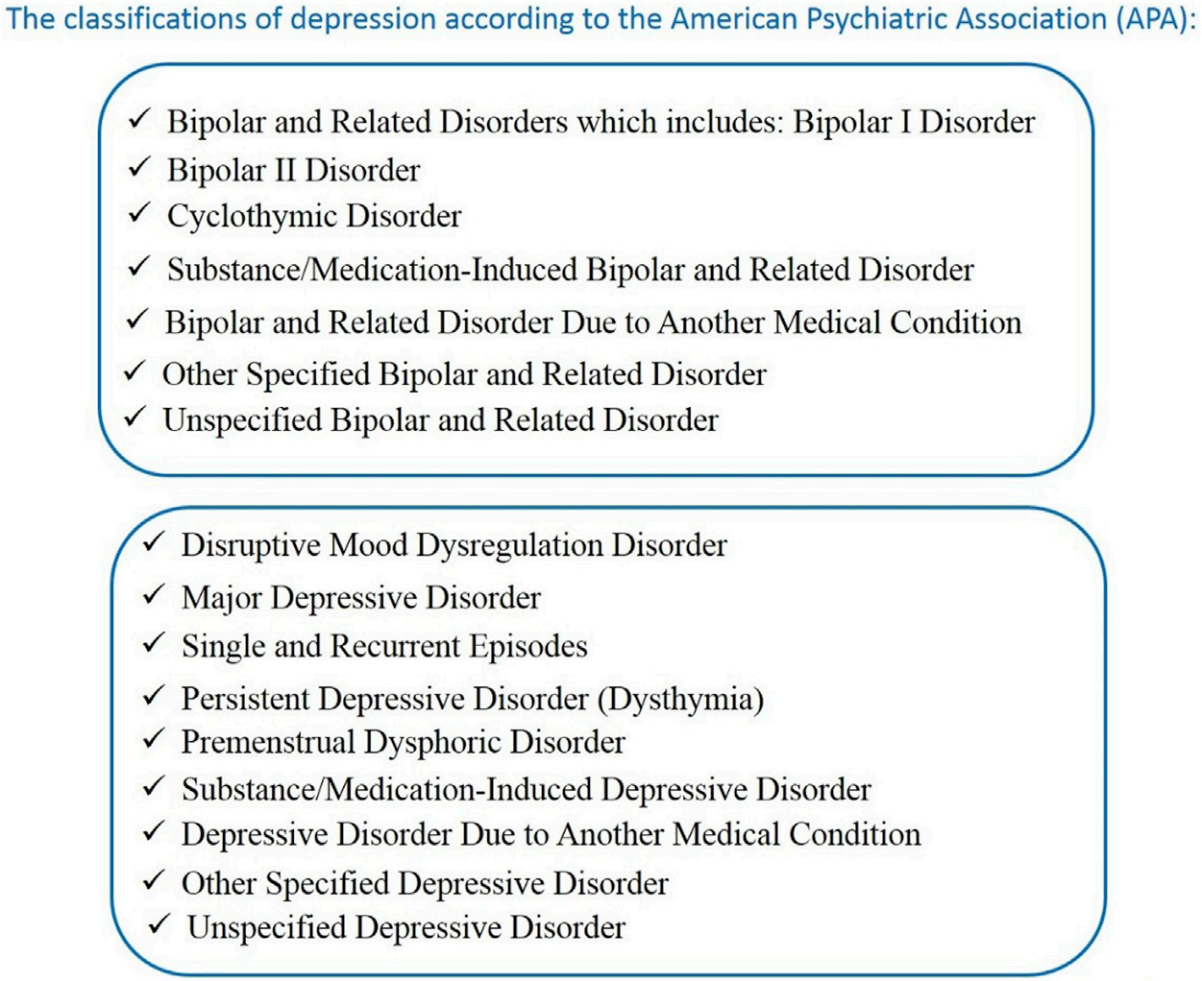
The classification of depression according to the American Psychiatric Association (APA). (DSM-5, 2013; Uher et al., 2014; Abdel-Bakky et al., 2021).

Following World Health Organization (WHO) data from the Institute of Health Metrics and Evaluation, the Global Health Data Exchange (GHDx) indicated that 280 million people in the world suffer from depression (Institute of Health Metrics and Evaluation Global Health Data Exchange, 2021). Depression is a very complex mental illness. Criteria for major depressive disorder (MDD) are one or more major depressive episodes (the lifetime absence of mania and hypermania) with five symptoms present during 2 weeks (DSM-5, 2013; Uher et al., 2014; Abdel-Bakky et al., 2021). The symptoms of depression can be divided into emotional and physical. The emotional symptoms of depression are stress, sadness, loss of interest, anxiety, hopelessness, difficulties with concentration, feeling of guilt, and suicidal thoughts (Abdel-Bakky et al., 2021). Physical symptoms include lack of energy, fatigue, pain, sleep disturbances, headaches, and psychomotor activity changes (Abdel-Bakky et al., 2021). The complexity of depression is evidenced by the classification of this illness proposed by the (American Psychiatric Association (APA), 2013) shown in Figure 1.

MDD can be divided into 14 subcategories, including that: “with anxious distress,” “with mixed features,” “with melancholic features,” “with mood-congruent psychotic features,” “with mood-incongruent psychotic features,” “with catatonia,” “with peripartum onset” categories (DSM-5, 2013).

As you can see, depression is a complex disease, which makes it challenging to diagnose unequivocally. According to statistics, women suffer from depression more often. It has been documented that depression in women occurs three times more often than in men. Hormonal aspects significantly impact the course of depression and treatment, which is particularly evident in postpartum depression (Kroska and Stowe, 2020). However, recent events related to the COVID-19 pandemic have shown increased incidence in all gender and age groups, including children (Bueno-Notivol et al., 2021; Hawes et al., 2021). Symptoms such as depression, anxiety, and cognitive impairment are considered to be the main symptoms of the post-acute COVID-19 syndrome (Mazza et al., 2022).

The etiology of depression has not yet been fully established and may involve genetic and environmental factors (Gaebel et al., 2017). Depression often coexists with other mental disorders (e.g., anxiety disorders and substance use disorders) and various somatic illnesses, including cardiovascular disease (e.g., hypertension, coronary artery disease), metabolic syndromes (e.g., diabetes), respiratory diseases (e.g., chronic obstructive pulmonary disease), various deficiencies (e.g., severe anemia), infections (e.g., tuberculosis, AIDS, influenza), collagen disorders, endocrine diseases (e.g., hypothyroidism, Cushing’s disease), and others (Dornquast et al., 2017; Abdel-Bakky et al., 2021). It's known that depression may induce somatic disorders and *vice versa*—the presence of chronic somatic conditions may lead to the development of mental diseases. Patients with somatic diseases have a higher risk of developing mental illness. On the other hand, in people with severe mental illness, the risk of developing somatic disorders is twice as high as in patients without psychiatric disorders. The data indicate that nearly 50% of patients experiencing mental disorders have clinically significant comorbid physical illnesses, which often go undiagnosed for extended periods (Dornquast et al., 2017; Steffen et al., 2020). Importantly, it is indicated that comorbidities are the most critical factor influencing the economic burden of depression. Hence, it is necessary to consider them in the treatment of depression (Steffen et al., 2020).

Between 2003 and 2005, in the European Union (EU), about 27% of adults were affected by a mental disorder (Wittchen et al., 2011). The following survey in 2011 detected 164.7 million people affected by mental health problems, which was about 38.2% of people (Wittchen et al., 2011). 2011 survey included 14 new disorders not included in the 2005 study (Wittchen et al., 2011) hence differences; but the number of affected people is enormous. Recent events related to the COVID-19 pandemic caused by the SARS-COV-2 virus have resulted in a significant increase in the incidence of depression in the population (Bueno-Notivol et al., 2021). A study by Bueno-Notivol et al. (2021) found a global estimated prevalence of depression in 2021 was seven times higher than in 2017, which is about a 25% increase. Authors searched for cross-sectional, community-based studies listed on PubMed or Web of Science from 1 January 2020, to 8 May 2020, that reported prevalence of depression (Bueno-Notivol et al., 2021). A random-effects model was used to estimate the pooled proportion of depression (Bueno-Notivol et al., 2021). Interestingly, the meta-regression observation showed that the prevalence of depression was independent of the percentage of women, mean age at baseline, response rate, or methodological quality (Bueno-Notivol et al., 2021), which suggests a severe global problem. Similar observations were documented in the United States after the pandemic of COVID-19 (Hawes et al., 2021). Another increase in depression can be expected in connection with the outbreak of the war in Ukraine in 2022 (Jain et al., 2022).

### The antidepressants used in the clinic

**FIGURE 2:**
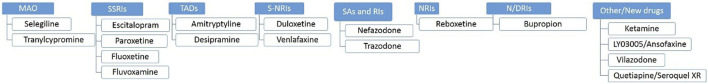
Selected examples of antidepressants by groups. MAO, Mono Amino Oxidase; SSRIs, Selective Serotonin Reuptake Inhibitors; TADs, Tricyclic Antidepressants; S-NRIs, Serotonin-Norepinephrine Reuptake Inhibitors; SAs and RIs, Serotonin Antagonist and Reuptake Inhibitors; NRIs, Norepinephrine Reuptake Inhibitors; N/DRIs, Noradrenaline and Dopamine Reuptake Inhibitors.

Today, the treatment of depression is primarily based on drugs discovered in the 1960s and 1970s. It should be mentioned here that Monoamine Oxidase Inhibitors (MAOIs) as selegiline, tranylcypromine, and phenelzine; Selective Serotonin Reuptake Inhibitors (SSRIs) as escitalopram, paroxetine, fluoxetine, fluvoxamine; Tricyclic Antidepressants (TADs) as amitriptyline, desipramine, iprindol; Serotonin-Norepinephrine Reuptake Inhibitors (S-NRIs) as venlafaxine, duloxetine, desvenlafaxine; Serotonin Antagonist and Reuptake Inhibitors (SAs and RIs) as nefazodone and trazodone; Norepinephrine Reuptake Inhibitors (NRIs) as reboxetine, viloxazine; Noradrenaline and Dopamine Reuptake Inhibitors (N/DRIs) as bupropion; and other drugs as esketamine, ansofaxine, vilazodone or quetiapine; see Figure 2. Moreover, just recently the FDA approved Zulresso (brexanolone) for postpartum depression (Powell et al., 2020).

The number and variety of antidepressants available today seem pretty large, but their use causes many problems. First, these drugs require a long administration time (except for esketmine) to obtain a therapeutic effect (Abdel-Bakky et al., 2021; McCarron et al., 2021). In addition, their use is associated with many side effects, such as weight gain, sexual dysfunction, dizziness, headache, anxiety, psychosis, cognitive dysfunctions, etc. (Abdel-Bakky et al., 2021; McCarron et al., 2021). Considering SSRIs, prolonged bleeding time, and, when used during pregnancy, heart defects and pulmonary hypertension in newborns have been observed (Berard et al., 2017; Calvi et al., 2021). For newer medications, such as those approved by the FDA for treatment of MDD, ansofaxine (potential triple reuptake inhibitor of serotonin, norepinephrine, and dopamine, approved in 2019) or vilazodone (a selective serotonin reuptake inhibitor and serotonin 5-HT1A receptor partial agonist, approved in 2021), the side effects are very similar to traditional antidepressants. They are mild to moderate, with a slightly different frequency than, for example, SSRIs or NRIs. Interestingly, sexual dysfunctions typical for conventional antidepressants are not observed after using ansofaxine (Mi et al., 2022; Chauhan et al., 2022). Also, esketamine, recommended for patients with drug-resistant depression, may have many unpleasant consequences, including dissociation, anxiety, nausea, increased blood pressure, and headache. These side effects are mild, transient, and dose-related and will disappear with subsequent treatments. It is also indicated that the frequency of their occurrence is twice as high in patients receiving simultaneously nasal ketamine and orally another antidepressant than esketemine alone (Ceban et al., 2021). Many of the drugs mentioned have not been approved for pregnant women (Abdel-Bakky et al., 2021; McCarron et al., 2021).

On the other hand, in patients with comorbidities, there are severe contraindications to combining antidepressants with other drugs due to possible drug-drug interactions. The risk of their occurrence is exacerbated by complex polypharmacy regimens and extended treatment periods which result in tolerance problems to ineffectiveness and serious adverse events (Low et al., 2018; Woron et al., 2019). Pharmacokinetic interactions of antidepressants and cardiac drugs seem particularly dangerous because cytochrome 450 isoenzymes metabolize both in the liver. Significantly, both groups of medications can affect the activity of these enzymes. For example, fluoxetine and paroxetine are inhibitors of CYP2D6, and calcium channel blockers inhibit the activity of CYP3A4. Consequently, combined drugs from both groups may lead to hypotension or an increased risk of gastrointestinal bleeding (Woron et al., 2019). Hence, when using polypharmacy, careful selection of drugs with a low interaction potential is necessary. Unfortunately, this is not always possible due to the limited availability of antidepressants with a simple metabolic profile. Moreover, in many cases (especially patients with the severe clinical condition), the possibility of oral administration of antidepressants is severely limited. The exception is esketamine, which the FDA has approved for treating drug-resistant depression as a nasal spray; its action is fast, but also be careful when using it due to the potential risk of drug-drug interactions (Turner, 2019).

All of this necessitates the search for new, more effective treatments for depression and mental health. First, there is a need for drugs that will give a quick therapeutic response, few side effects, and a limited amount of interactions with other medications. Therefore, in the next chapter, we will consider new strategies for treating depression and identify potential pharmacological targets for new active substances designed to treat depression effectively.

## Towards better treatment of depression

### The promising pharmacological targets

When looking for a new way to treat depression, it is essential to define the main cellular/molecular targets for these findings. At present, the most important new targets for antidepressants seem to be glutamate receptors (GluRs) (Stachowicz 2021a; Stachowicz, 2021; Vasiliu 2022a) or gamma-aminobutyric acid (GABA)-ergic modulators; paying attention to both intra- and extra-synaptic GABA-A receptors (Brickley and Mody, 2012; Luo et al., 2013; Vasiliu, 2022). Although the use of GABA-ergic ligands seems to be effective in the treatment of depression, direct interference with the GABA pathway has side effects in the form of drowsiness or sedation, which may hinder daily activities (Vasiliu, 2022); for this reason, the weight appears to be tipping in favor of the GluRs ligands. These agents seem to be an auspicious new point in the pharmacological treatment of depression (Stachowicz 2021a; Stachowicz, 2021; Vasiliu 2022a), which is confirmed by the scale of both preclinical phase I and III clinical trials, where we can find as many as 27 active substances following the last finding (Stachowicz 2021a; Stachowicz, 2021; Vasiliu 2022a). Among GluRs ligands considered in clinical use were NMDA receptor (NMDAR) or its GluN2A/GluN2B subunits antagonists (e.g., NRX-101 or AZD6765, respectively), NMDAR positive allosteric modulators (e.g., AGN-241751), NMDAR-glycine site agonists (e.g., GLYX-13), AMPA receptor potentiators (e.g., TAK-653) and metabotropic glutamate receptors (mGluRs) antagonists (e.g., TS-161) (Stachowicz 2021a; Vasiliu 2022a). Among them, AZD6765, GLYX-13, and TAK-653 did not reach clinical use (Kadriu et al., 2020). However, compounds like GLYX-13 became the prototype for the synthesis of the next generation of compounds with similar mechanisms of action, including apimostinel (GATE-202, NRX-1074), a second-generation analog with increased potency, and zelquistinel (GATE-251, AGN-241751), a third-generation small molecule with increased power and high oral availability (Neurosciences Gate, 2022). The central hypothesis in using these compounds in depression is based on the idea that excessive excitatory transmission related to Glu and so-called neurotoxicity lead to impairment of brain functions, transmission, and plasticity, manifested by mental disorders such as depression (Skolnick, 1999; Stachowicz 2021a).

Other groups of active substances considered in the pharmacotherapy of depression are not apparent compounds acting as anti-cytokines and COX-2 inhibitors (Stachowicz 2021a; Vasiliu 2022a). This group of active substances is aimed at patients with immunological disorders coexisting with depression, where it achieves impressive results both as monotherapy and in combination with classical antidepressants (Stachowicz 2021a; Vasiliu 2022a). Importantly, these compounds could be effective in patients after COVID-19 infection because the psychopathological mechanisms underlying the symptoms of depression after COVID-19 are mainly related to inflammation caused by the peripheral immune response to viral infection and persistent psychological stress during and after an illness. Currently, eight active substances are in phase I to IV clinical trials (Vasiliu 2022). However, clinical reports are already showing promise with combination therapy for COX-2. Following Sethi et al. (2019), the favored use of celecoxib with reboxetine, fluoxetine, and sertraline was observed in depressed patients. In the latter, improvements in HRDS and HAM-D scales were observed if antidepressants were combined with celecoxib. Our preclinical studies are also optimistic, as positive antidepressant effects were observed in the animal model after co-administration of COX-2 inhibitor (NS398) with mGluR ligand and imipramine (Stachowicz 2021a). We have also started to decipher the mechanisms of the influence of antidepressants on fertility parameters in rodents, which may be crucial when looking for active substances without side effects (Solek et al., 2021; Tabecka-Lonczynska et al., 2021).

**FIGURE 3:**
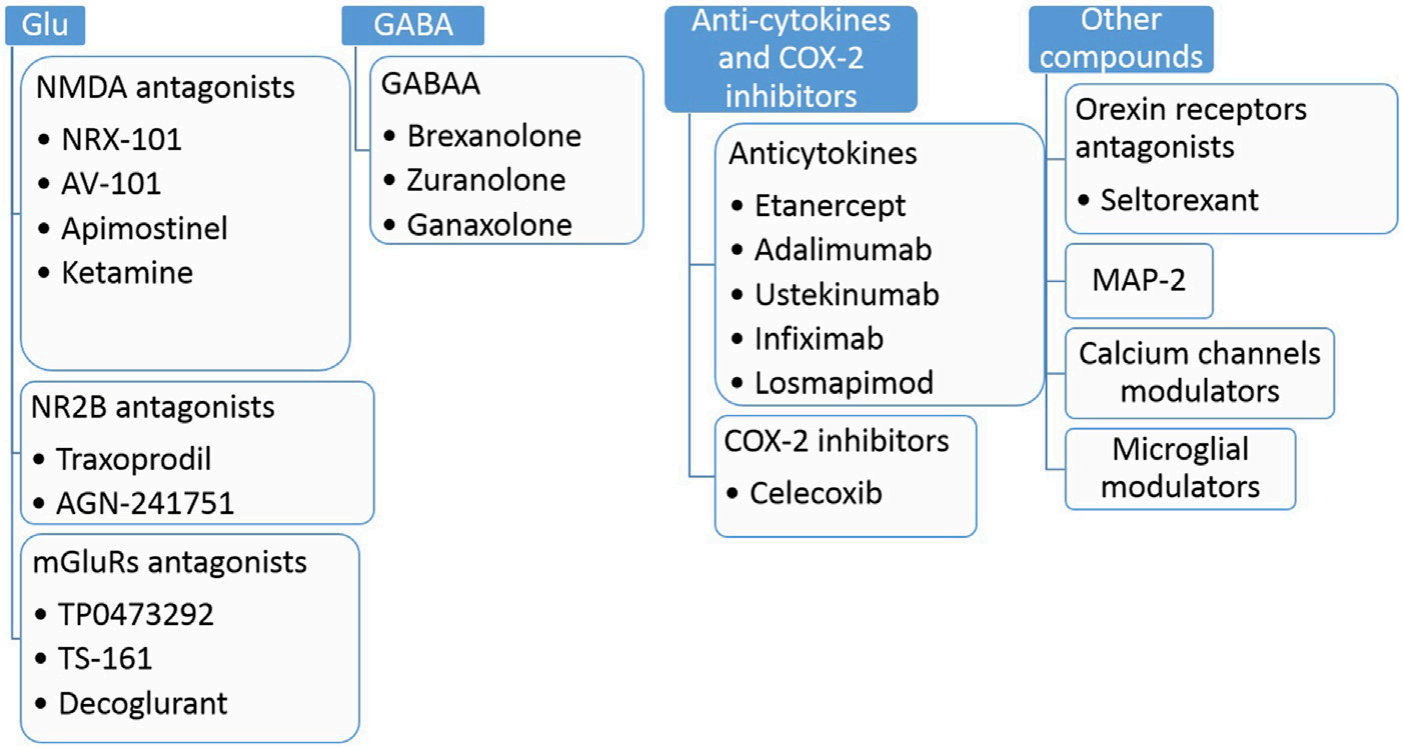
Selected examples of antidepressant compounds in clinical trials (Phase I-IV). Glu, glutamate; NMDA, N-methyl-D-aspartate; NR2B, NMDA receptor subunit 2B; mGluRs, metabotropic glutamate receptors, GABAA, gamma-aminobutyric acid receptors A; COX-2, cyclooxygenase 2; MAP-2, microtubule-associated protein 2.

Quite a new group of antidepressants are orexin receptor antagonists, or compounds acting through microtubule-associated protein type-2 (MAP-2) *via* calcium channels or microglial mechanisms (for a more extensive review on the topic, please refer to Vasiliu 2022; Vasiliu 2022a). As far as orexins are concerned, ligands for type 1 and 2 receptors (OX1R and OX2R) can modulate feeding, sleep, motivated behavior, anxiety, and addiction; hence they have a vast potential to regulate many aspects of depression (Vasiliu 2022). For details described in this section, see Figure 3.

Of course, we must not forget the substances that affect the 5-HT system. Intensive work is underway in this field to implement new compounds acting through new receptors such as 5-HT7 or safer compounds directed towards 5-HT1A or 5-HT2A (e.g., MIN-117 or psilocybin, respectively) (Vasiliu 2022); the topic will be expanded in the following subsections.

### Combination therapy

**FIGURE 4:**
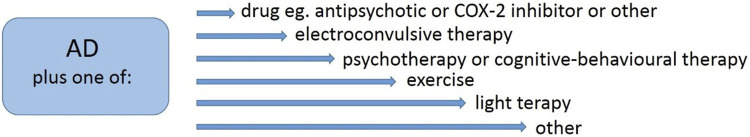
Examples of combined therapy in the treatment of depression. AD, antidepressant; COX-2, cyclooxygenase 2.

Because it was estimated that 15%–20% of depressed patients do not respond to the treatment (Rush et al., 2006; Kalmoe et al., 2020), there are ongoing attempts to use combination therapy to improve mental health (Figure 4).

One way to improve the outcome of the treatment is a combination of known antidepressants (ADs) with the substance directed to a different target, e.g., combination with antipsychotics (Davis et al., 2021; Vázquez et al., 2021; Kishi et al., 2021) or COX-2 inhibitors (Muller et al., 2006; Muller, 2019; Sethi et al., 2019; Stachowicz, 2021). Second-generation antipsychotics (e.g., olanzapine, risperidone, or aripirazole) are effective in combination therapy; however, they still have not overcome the effectiveness of combination therapy with lithium (Vázquez et al., 2021; Kishi et al., 2021). Some hope is linked with combination therapy with COX-2 inhibitors, while much evidence is that this path is effective (Muller et al., 2006; Muller, 2019; Sethi et al., 2019).

A combination of electroconvulsive therapy with ADs is known, and the recent discovery regarding its use with esketamine seems very promising and demonstrates high efficacy in drug-resistant depression (Kavakbasi et al., 2021). Subsequent directions of combining treatments focus on ADs administration and application of psychotherapy (Guidi and Fava, 2021), cognitive-behavioral therapy with virtual reality (Stamou et al., 2021), exercise (Xie et al., 2021), or light therapy (Even et al., 2008) and others. These methods enrich the treatments that benefit and require specialists’ involvement and the patient’s engagement.

The last decade’s new finding is a microbiota-gut axis in depression (Cryan et al., 2019; Stachowicz, 2019; Simpson et al., 2021). Following Cryan et al. (2019), there is a new concept: “the concept of psychobiotics for treating various neurological and psychiatric disorders through targeting the gut microbiota.” The idea of enriching therapy of depression by psychobiotics directed research into entirely new paths; hence a vast amount of research in laboratories around the world is moving in this direction (Cryan et al., 2019; Stachowicz, 2019; Simpson et al., 2021). In connection with the above, mutual regulation between host microbiota and the effectiveness of ADs was recently described (Cryan et al., 2019; Stachowicz, 2019; Duan and Xie, 2020; Simpson et al., 2021); the topic will be expanded in the last subsection.

The latest findings of the COVID-19 pandemic and social isolation documented the positive effects of gardening and physical activity on mental health (Bu et al., 2021). The benefits of gentle exercises for mental health may be connected with reducing blood pressure, regulating neuroendocrine and neuroimmune systems, and giving psychological benefits (Bu et al., 2021).

### A new look at old drugs

When presenting new ideas for treating depression, one cannot ignore the idea of a “new look at old drugs.” Compounds that affect the 5-HT system are a large group of AD in the clinic, including 5-HT1A agonists, e.g., vilazodone and vortioxetine (Wróbel et al., 2019). Searching for new antidepressant compounds is focused on that directed to dual agonist 5-HT1A activity and SSRI (Herold et al., 2011; Wróbel et al., 2019) as a compound that both can accelerate desensitization and downregulation of autoreceptors and directly stimulate postsynaptically localized 5-HT neurons (Herold et al., 2011; Wróbel et al., 2019). The idea is that kind of stimulation has a faster antidepressant potential. Among dually acting compounds, molecular targets are 5-HT2A, 5-HT6, 5-HT7, and D2 (Wróbel et al., 2019). Analogs of gepirone, novel pyridol/pyrimidine derivatives, alkylnitroquipazines, and others are synthesized (Paluchowska et al., 2005; Herold et al., 2011; Wróbel et al., 2019; Ślifirski et al., 2019; Król et al., 2021).

Inhibitors of the serotonin transporter (SERT) have long been in clinical use. The “new look at old drugs” regarding SERT compounds is based on the physicochemical properties of SERT ligands. Understanding of physicochemical properties of interactions with targeted sites may be beneficial in designing new compounds with antidepressant properties. Does conformational rearrangement or ligand flexibility play a role in binding reaction and efficacy (Martin et al., 2008)? Martin et al. (2008) documented increased enthalpy with a polar surface area of S-citalopram, duloxetine, fluoxetine, indatraline, paroxetine, sertraline, venlafaxine, but not fluvoxamine; what suggests a SERT inhibitor binding site is polar and allows hydrated ligands to bind without imposing the enthalpic penalty expected from ligand dehydration (Martin et al., 2008). Furthermore, following Martin et al. (2008), entropy/enthalpy compensation in ligand-protein interactions is counteracted by the following regulation (conformational flexibility or hydrophobic properties are regulators of entropy H-bonds or van der Waals interactions in enthalpy). Martin's group found SERT inhibitors bind to 5-HT transporter in a competitive manner (Apparsundaram et al., 2008), further concluding that 5-HT pore may allow for more than one set of interactions with antidepressants (Apparsundaram et al., 2008).

Antipsychotics have attracted some attention in the last decade; the more it has been observed that discontinuation of antidepressants correlates with hypomania or mania (Kassm and Naja, 2018). Second-generation antipsychotics, e.g., aripiprazole, quetiapine, and olanzapine in combination with fluoxetine, have been approved to treat depression (Kato and Chang, 2013). The target audience is patients who failed to respond to monotherapy with ADs (Kato and Chang, 2013). However, Second-generation antipsychotics were more potent than placebo; esketamine or lithium use trumped the antipsychotic results (Vázquez et al., 2021). The research is ongoing, so there is no conclusion.

### The new ideas and directions of (preclinical) research

This subsection would not be presented with accepted and established trends, e.g., the antidepressant effects when targeting trophic factors like brain-derived neurotrophic factor (BDNF) or results of drugs directed to tropomyosin receptor kinase B (TrkB). Here, there will be many new ideas born in the depression field.

One fresh concept is described in Stachowicz (2018) and Wong et al. (2013). Rearrangement of the cytoskeleton of dendrites and spines and adhesion between spines as a predictor of mental health is an entirely new research direction. Following Wong et al. (2013), cytoskeletal abnormalities cause dendritic regression and decrease and are common in depression. In cytoskeletal rearrangement, actin filaments are engaged (F-actin, G-actin) and also actin-binding proteins (ABPs) and postsynaptic density (PSD) proteins, creating an interactive dendritic spine scaffold (Wong et al., 2013). The interplay of actin filaments with microtubules is responsible for organelles’ circulation in the dendritic spine and thus for the rotation of the receptor components and anchoring them in the cell membrane (Wong et al., 2013). Various types of receptors are present in the PSD. Still, the ionotropic glutamate receptors (iGluRs) seem particularly important in depressive disorders and the search for new anti-depressants. For a long time, it was thought that a sufficient explanation of the functional changes in the excitatory transmission is post-translational modifications (e.g., phosphorylation) located in the postsynaptic membrane receptors. However, the studies from the last 30 years have destabilized the static image of excitatory synapses and revealed its highly dynamic structure. It is known that glutamate receptors show lateral mobility along the cell surface between synaptic and extrasynaptic regions. They undergo constitutive trafficking to and from the cell surface with a surface half-life measured in 10 min (Nishimune et al., 1998). They are delivered to and removed from the synaptic membranes regulated by neural activity or the degree of electrical stimulation of the neuron. These mechanisms control the number of receptors and the receptors’ subunit composition, which determines the proper functioning of excitatory synapses. Regulation of the movement of receptors to synapses is multistep and includes transport from the endoplasmic reticulum, trafficking along dendrites, and local transport in a synaptic bulb. This process is controlled by numerous PSD proteins interacting with receptors *via* PDZ domains (e.g., PSD-95, Shank3/ProSAP2, SAP-97, PICK-1), and other scaffolding proteins (e.g., Homer, CaMKII) and the same receptors, which include many sites undergoing (not only) phosphorylation. Significantly, PSD proteins interact with many intracellular proteins (e.g., PSD-95 with synaptic Ras GTPase activating protein and guanylate kinase-related protein) (Dutta et al., 2021; Shaw and Koleske, 2021).

In addition to the above-discussed processes and molecules, adhesion is essential for synaptic formation by associating pre- and post-synaptic partners in a specific neuro space (Stachowicz, 2018). The importance of the problem was noted by Stachowicz (2018) in the review of the DSCAM protein. DSCAM is only an example, but the adhesive mechanisms involving other adhesive proteins are fundamental in synaptic plasticity and, thus, neural conduction and communication (Stachowicz, 2018; Jiang et al., 2021). Abnormal synaptic connection is associated with learning and memory disturbances and neuropsychiatric and neurodevelopmental disorders (Dutta et al., 2021).

An entirely new finding in the field of depression is the discovery of a new signal pathway involved in the disease—that is, engagement of the antioxidant pathway with nuclear factor erythroid-derived 2-like 2 (Nrf2) (Kansanen et al., 2013; Bouvier et al., 2017; Nakayama et al., 2020). Nrf2 was discovered in 1994 as a member of the human cap“n”collar (CNC) basic-region leucine zipper transcription factor family (Cuadrado et al., 2019). As a product of the NFE2L2 gene, Nrf2 forms heterodimers with other bZip proteins; and regulates the expression of about 250 human genes participating in inflammation, redox metabolism, or proteostasis (Robledinos-Anton et al., 2019). Activators of the Nrf2 pathway are under clinical investigation in Phase I-IV in multiple sclerosis, autism spectrum disorder, Alzheimer’s disease, major depression, and others (Robledinos-Anton et al., 2019). In a depression field, activation of Nrf2 translocation restores redox homeostasis and reverses vulnerability to depression (Bouvier et al., 2016). Furthermore, Nrf2-null mice show depressive-like behavior, and treatment with Nrf2 agonists possesses antidepressant-like potential (Nakayama et al., 2020).

In recent years another discovery in the field of depression is the already mentioned microbiota-gut axis (Cryan et al., 2019; Stachowicz, 2019; Simpson et al., 2021). Our results with the *E. coli* lipopolysaccharide (LPS) use suggest the engagement of this mechanism in synaptic plasticity with the involvement of excitatory amino acid transporters (EAATs)/COX-2/metabotropic glutamate receptors (mGluRs) (Stachowicz et al., 2021). However, more sophisticated research documented the gut microbiota may regulate the hypothalamic-pituitary-adrenal (HPA) axis, producing neurotransmitters (Sirisinha 2016). Probiotics containing appropriate species of bacteria can lower cortisol; microbiota transplanted from a healthy animal can change animal behavior (Sirisinha 2016). This search for mechanisms of depression, thus related to the immune system, also includes recent reports on the occurrence of depression after COVID-19. Following Mohammadkhanizadeh and Nikbakht (2021), central mechanisms involved in COVID-19-induced depression are inflammation, including uncontrolled activation of microglia, and following the release of inflammatory cytokines (TNF-alpha, IL-6, IL-1beta), nitric oxide, prostaglandin E2. Furthermore, damage to mitochondria directly by reaching them for transcription of the virus genome and indirectly by devasting properties of pro-inflammatory cytokines and ROS (Mohammadkhanizadeh and Nikbakht, 2021). Damage to the hippocampus observed after COVID-19 as a structure involved in depression has drawn the particular attention of researchers to the mechanisms linking respiratory viral infections and depression (Mohammadkhanizadeh and Nikbakht, 2021). Impairment of hippocampal synaptic plasticity and neurogenesis, followed by stress and dysregulation of the HPA axis, contributes to the progression of symptoms of depression (Mohammadkhanizadeh and Nikbakht, 2021). Next, vitamin D deficiency, Zinc, and magnesium are related to depression in COVID-19 (Mohammadkhanizadeh and Nikbakht, 2021).

Inflammatory processes in depression have long been a focus of research. Elevated levels of pro-inflammatory cytokines such as IL-1b, IL-6, and TNFa are observed in the serum of patients with depression (Iwata et al., 2013). The cytokines above are associated with somatic symptoms, described as illness behaviors, including fatigue and loss of appetite (Iwata et al., 2013), which overlap with typical symptoms of major depression. Subsequently, depression has been well described as a common complication of interferon treatment for malignant melanoma and chronic hepatitis (Musselman et al., 2001), and interferon-induced depression has been linked to a complex pathophysiological substrate involving serotonergic and dopaminergic neurotransmission as well as glucocorticoid and neurotrophic factors (Udina et al., 2016).

## Concluding remarks

Summing up, as it can be seen based on this study, there is an intense search for new substances with an antidepressant profile. Researchers have focused on the hunt for compounds that directly interfere with the functioning of the Glu system through its various receptors, both NMDA, AMPA, and mGluRs. In addition, the next direction of research is still the 5-HT system. Still, in a new perspective, the game includes new receptors, such as 5-HT7, and multidirectional therapy, such as, e.g., triple reuptake inhibitors of 5-HT/norepinephrine/dopamine. These are, of course, the main directions of the search. Still, there are also attempts to search for active substances among compounds acting by orexin receptors, COX-2 inhibitors, incorporation of phagocytic, microglial, epigenetic mechanisms, or combination therapies. Personalized antidepressant treatment should also be considered in the future, considering gender differences or genetics, among other things. This problem was described in 2012 by Gvozdic et al. (2012) Undoubtedly, the development of new antidepressants based on new mechanisms of action is necessary due to the increasing number of patients, the unsatisfactory effectiveness of existing pharmacotherapy, or possible side effects and interactions with drugs used for other diseases.
